# Miscellany

**Published:** 1901-12

**Authors:** 


					﻿LITERARY NOTES.
The American Journal of the Medical Sciences, so long- recog-
nised as the leading monthly medical magazine in this country,
has enlarged its space allotted to original communications, so
that its November, 1901, issue contained double that usually set
apart for the department referred to. The enlargement will be
permanent. This magazine, always noted for its high-class origi-
nal articles, abstracts, and reviews, will continue its energies in
these directions, aiming in the future, as in the past, to stand in
the front rank of medical periodicals.
W. B. Saunders & Co., publishers, Philadelphia, announce the
publication of Nothnagel’s Encyclopedia of practical medicine,
edited by Dr. Alfred Stengel, professor of clinical medicine in the
University of Pennsylvania. The set will consist of ten or
twelve volumes, translated by competent men who know English
and German thoroughly, and who are acquainted with the sub-
jects which they translate. Five or six volumes will appear
during 1902 and the remainder soon afterward.
The usual method of publishers, when issuing a work of this
kind, has been to compel physicians to take the entire system.
This seems to us in many cases to be undesirable. Therefore, in
purchasing this encyclopedia, physicians will be given the oppor-
tunity of subscribing for the entire system at one time; but any
single volume or any number of volumes may be obtained by
those who do not desire the complete series. This latter
method, while not so profitable to the publisher, offers to the
purchaser many advantages which will be appreciated by those
who do not care to subscribe for the entire work at one time.
This American edition of Nothnagel’s Encyclopedia will, with-
out question, form one of the greatest systems of medicine ever
produced, and the publishers feel confident that it will meet with
general favor in the medical profession.
LITERARY NOTES.
The American Journal of the Medical Sciences, so long- recog-
nised as the leading monthly medical magazine in this country,
has enlarged its space allotted to original communications, so
that its November, 1901, issue contained double that usually set
apart for the department referred to. The enlargement will be
permanent. This magazine, always noted for its high-class origi-
nal articles, abstracts, and reviews, will continue its energies in
these directions, aiming in the future, as in the past, to stand in
the front rank of medical periodicals.
W. B. Saunders & Co., publishers, Philadelphia, announce the
publication of Nothnagel’s Encyclopedia of practical medicine,
edited by Dr. Alfred Stengel, professor of clinical medicine in the
University of Pennsylvania. The set will consist of ten or
twelve volumes, translated by competent men who know English
and German thoroughly, and who are acquainted with the sub-
jects which they translate. Five or six volumes will appear
during 1902 and the remainder soon afterward.
The usual method of publishers, when issuing a work of this
kind, has been to compel physicians to take the entire system.
This seems to us in many cases to be undesirable. Therefore, in
purchasing this encyclopedia, physicians will be given the oppor-
tunity of subscribing for the entire system at one time; but any
single volume or any number of volumes may be obtained by
those who do not desire the complete series. This latter
method, while not so profitable to the publisher, offers to the
purchaser many advantages which will be appreciated by those
who do not care to subscribe for the entire work at one time.
This American edition of Nothnagel’s Encyclopedia will, with-
out question, form one of the greatest systems of medicine ever
produced, and the publishers feel confident that it will meet with
general favor in the medical profession.
MISCELLANY.
Prize for Original Research in Epilepsy.—Dr. Frederick
Peterson, New York State Commissioner of Lunacy, has offered
a prize of $200 for the best original unpublished contribution
to the pathology and treatment of epilepsy. All manuscript
should be submitted.in English, and the prize isopen to universal
competition. Each essay must be accompanied by a sealed
envelope, containing the name and address of the author and
bearing- upon the outside a motto or device, which is to be
inscribed also upon the essay. All papers received will be sub-
mitted to a committee, consisting- of three members of the New
York Neurolog-ical Society, and the award will be made upon
its recommendation at the annual meeting- of the board of
managers of the Craig Colony, October 14, 1902. Manuscripts
should be sent to Dr. Frederick Peterson, 4 West Fiftieth Street,
New York, on cr before September 30, 1902. The successful
essay becomes the property of the Craig Colony and will be
published in its medical reports.
				

